# Irisin and Secondary Osteoporosis in Humans

**DOI:** 10.3390/ijms23020690

**Published:** 2022-01-08

**Authors:** Roberta Zerlotin, Angela Oranger, Patrizia Pignataro, Manuela Dicarlo, Filippo Maselli, Giorgio Mori, Silvia Concetta Colucci, Maria Grano, Graziana Colaianni

**Affiliations:** 1 Department of Emergency and Organ Transplantation, University of Bari, 70124 Bari, Italy; r.zerlotin@gmail.com (R.Z.); angelaoranger@yahoo.it (A.O.); 2 Department of Basic Medical Sciences, Neuroscience and Sense Organs, University of Bari, 70124 Bari, Italy; patrizia.pignataro@uniba.it (P.P.); manuela.dicarlo@uniba.it (M.D.); silviaconcetta.colucci@uniba.it (S.C.C.); 3 Department of Neurology, Rehabilitation, Ophthalmology, Genetics, Maternal and Child Health (DINOGMI), University of Genoa, 16132 Genoa, Italy; masellifilippo76@gmail.com; 4 Department of Clinical and Experimental Medicine, University of Foggia, 71100 Foggia, Italy; giorgio.mori@unifg.it

**Keywords:** irisin, osteoporosis, hyperparathyroidism, Prader–Willi syndrome, growth hormone, Cushing’s disease, inflammatory bowel disease

## Abstract

Irisin is a peptide secreted by skeletal muscle following exercise that plays an important role in bone metabolism. Numerous experiments in vitro and in mouse models have shown that the administration of recombinant irisin promotes osteogenesis, protects osteocytes from dexamethasone-induced apoptosis, prevents disuse-induced loss of bone and muscle mass, and accelerates fracture healing. Although some aspects still need to be elucidated, such as the dose- and frequency-dependent effects of irisin in cell cultures and mouse models, ample clinical evidence is emerging to support its physiological relevance on bone in humans. A reduction in serum irisin levels, associated with an increased risk of osteoporosis and bone fractures, was observed in postmenopausal women and in both men and women during aging, Recently, cohort studies of subjects with secondary osteoporosis showed that these patients have lower circulating levels of irisin, suggesting that this myokine could be a novel marker to monitor bone quality in this disease. Although there are still few studies, this review discusses the emerging data that are highlighting the involvement of irisin in some diseases that cause secondary osteoporosis.

## 1. Introduction

Osteoporosis is a progressive multifactorial skeletal disorder characterized by the deterioration of bone microarchitecture and increased susceptibility to fracture risk [1].

The increase in life expectancy, causing a higher average age of the population, as the main achievement of modern science, consequently leads to an increase in the incidence of chronic diseases typical of the elderly, such as osteoporosis [1]. Typically, the elderly population with osteoporosis is often concomitantly affected by sarcopenia, which progressively leads to a loss of muscle mass and strength, thus amplifying the risk of fractures [2]. Osteoporosis and sarcopenia are the most common musculoskeletal disorders in the elderly; however, they can also affect young people with metabolic disorders, neurodegenerative diseases, cancer diseases, and astronauts during space missions due to weightlessness. Osteoporosis and sarcopenia represent a dangerous “duet” with a significant social relevance for the great socio-health impact of the consequent fractures. Pharmacologically, while some measures to treat sarcopenia are beneficial for bone health, the treatment of osteoporosis does not always reflect positively on muscles. Regular exercise is one of the proven non-pharmacological strategies to prevent bone fragility and sarcopenia. However, not all individuals are in a condition to perform regular physical activity; therefore, the identification of exercise-mimicking molecules represents a resource to prevent and/or treat both diseases.

**Figure 1:**
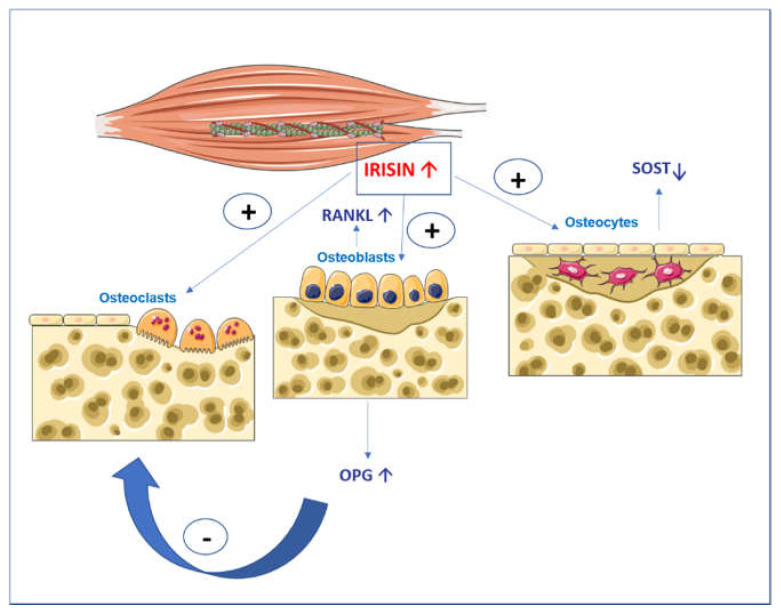
Graphical illustration of the action of irisin on bone cells. Irisin increases osteoblast differentiation and activity and affects osteocytes by increasing their viability and inhibiting the expression of Sost, the gene coding for sclerostin. Irisin has a double action on osteoclasts: an indirect action through the increase in osteoprotegerin (OPG) expression in osteoblasts that block the receptor activator of nuclear factor kappa-Β ligand (RANKL), and in parallel, a direct action by stimulating the differentiation of osteoclast precursors.

Myokine irisin is a protein secreted into the blood by cleavage of membrane protein 5 (FNDC5) after a skeletal muscle contraction under the control of coactivator 1-alpha (PGC1alpha) [3]. Early studies by Bostrom et al. showed the effect of irisin in activating the trans-differentiation of white adipose tissue into brown tissue. Irisin was found to play important roles in metabolic disorders, Alzheimer’s disease, brain function, and bone metabolism [4,5,6]. Several studies demonstrated that irisin influences bone cells [7,8,9,10,11,12,13]. Specifically, it was shown that irisin stimulates osteoblast differentiation and activity, through the upregulation of transcription factors and matrix proteins such as the Activating Transcription Factor 4 (Atf4) and Collagen I. In addition, irisin directly affects osteocytes by increasing their viability. In parallel, irisin has a dual action on osteoclasts: an indirect action through the increased expression of Osteoprotegerin (OPG) by osteoblasts [8,11] and a direct action in stimulating osteoclastogenesis of osteoclast precursors treated continuously with 10 ng/mL of recombinant irisin (rec-irisin) (Figure 1) [12].

However, the same authors observed that a higher dose (20 ng/mL) increased the number of osteoclasts significantly less than the 10 ng/mL dose, and doses of irisin equal to or greater than 100 ng/mL decreased osteoclastogenesis [12]. Furthermore, Zhang and colleagues [14] treated pre-osteoclastic RAW264.7 cells with rec-irisin for 3 days and observed a significant reduction in the mRNA levels of tartrate-resistant acid phosphatase (TRAP) nuclear factor of activated T cells (NFATc1), and cathepsin K (CatK) [14]. In addition, the difference observed in vivo between the study by Estell et al. [12] and the study by Zhang et al. [14], may be due to the duration of irisin treatment, i.e., short duration (7 days) promoting osteoclastogenesis [12], and chronic treatment (2 months) [14] inhibiting osteoclastogenesis through the increased activity of the Mck promoter of Fndc5.

**Figure 2:**
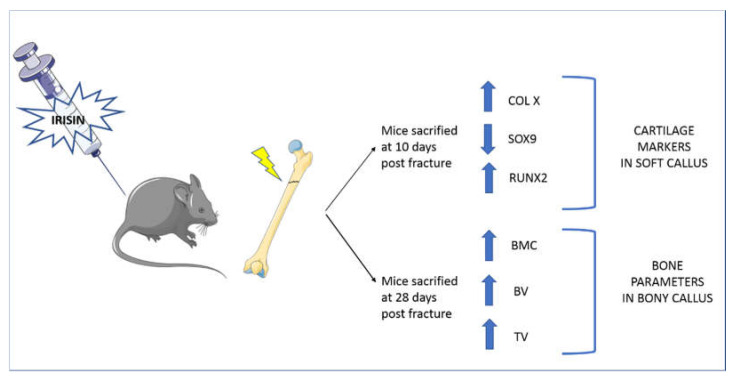
Systemic administration of recombinant irisin accelerates fracture healing in mice. Treatment with irisin administered at a low dose (100 μg/kg) and intermittently (once a week) increased X-type collagen expression in the cartilaginous callus at 10 days after fracture, indicating a more advanced stage of endochondral ossification of the callus during the early phase of fracture repair. Further evidence that irisin induced the transition of cartilaginous callus into osseous callus was provided by a reduction in SRY (sex-determining region Y)-box 9 (SOX9) and an increase in runt-related transcription factor 2 (RUNX2). At 28 days after fracture, microCT analyses showed that total callus volume (TV), bone volume (BV), and bone mineral content (BMC) were increased in irisin-treated mice compared with controls.

Therefore, it was hypothesized that irisin concentrations, as well as frequency and duration of treatment, are responsible for the discrepancies observed in the different studies. Experiments conducted in vivo on young healthy mice show a positive effect of rec-irisin on cortical bone and its mechanical properties, improving some parameters such as cortical bone surface, tissue mineral density, cortical perimeter and polar moment of inertia, an index of resistance of long bone to torsional forces [10]. Follow-up studies on osteoporotic mouse models showed that treatment with rec-irisin prevented both cortical and trabecular bone mineral density (BMD) reduction in mice subjected to four weeks of unloading [11]. Furthermore, if irisin was administered after four weeks of unloading, when bone loss already occurred, cortical and trabecular BMD loss were reverted, indicating the potential of irisin to also treat osteoporosis [11]. In contrast with these results, Kim and colleagues showed that mice with a global deletion of the irisin precursor, FNDC5, were resistant to ovariectomy-induced bone loss through the inhibition of osteoclastic bone resorption and osteocytic osteolysis [13]. The authors also observed an increased expression of sclerostin, an inhibitor of bone formation, after 6 daily injections of 1 mg/kg of irisin [13]. In contrast, a reduction in sclerostin was observed by injecting unloaded mice with a 10 times lower dose, given weekly for 4 weeks [11]. Similar to the parathyroid hormone (PTH), which exerts both catabolic and anabolic effects on the skeleton depending on the administration regimen [15], it was hypothesized that a high dose of irisin could lead to bone catabolism [13], whereas a lower dose, given with intermittent pulses of irisin, as occurs during exercise, could have anabolic effects on bone [11]. To further explore this hypothesis, studies were conducted to evaluate the effects of irisin on osteocyte viability when it was administered at low doses and intermittently, as occurs during exercise. The results showed that the treatment of unloaded mice with 100 μg/kg weekly of rec-irisin for four weeks inhibited disuse-induced osteocytes apoptosis and reduced the number of empty lacunae compared to unloaded mice treated with a vehicle [16]. In vitro studies were conducted on osteocyte-like cell lines (Mlo-y4), demonstrating that irisin treatment increases osteocyte survival by upregulating Blc2/Bax ratio and preventing dexamethasone and hydrogen peroxide-induced caspase activation. Moreover, in vivo studies also showed an inhibition of caspase activation in the cortical bone of unloaded mice treated with rec-irisin [16]. Additionally, rec-irisin activated the MAP kinases, Erk1 and Erk2, and increased the expression of the transcription factor Atf4 through an Erk-dependent pathway in osteocytes [16]. These results revealed the basic mechanisms of irisin’s action on osteocytes; to increase their functions and exert antiapoptotic effects, confirming that mechanosensory cells in bone are sensitive to the exercise-mimetic myokine irisin [16]. Very recently, it was shown that the systemic administration of an intermittent, low dosage of irisin accelerates bone fracture healing in mice [17]. By examining the impact of irisin treatment after 10 and 28 days post fracture, we observed an accelerated shift of cartilage callus to bony callus, along with a modification of chondrocytes towards the hypertrophic phenotype, and an increase in callus volume and bone mineral content, indicating a more rapid mineralization without affecting trabecular architecture and bone remodeling (Figure 2) [17].

In support of the importance of irisin in the human musculoskeletal system, observational studies have shown that circulating irisin levels correlate positively with parameters of healthy bone and muscle tissues [18,19]. Recently, we described a positive correlation between serum irisin and both femoral and vertebral bone mineral density in a population of elderly subjects [20]. Levels of the irisin precursor, FNDC5, in skeletal muscle of these subjects correlated positively with serum irisin levels and osteocalcin expression in bone biopsies, indicating a strong correlation between muscle and bone [20]. In the same study, we provided in vitro evidence demonstrating that treatment with rec-irisin in osteoblasts reduces the expression of p21, one of the effectors of the senescence process [20]. Therefore, these results suggest that this molecule could represent a viable therapeutic option to delay osteoporosis caused by senescence [21].

All these in vitro and in vivo studies demonstrated the importance of irisin action on bone metabolism. Although some aspects remain to be elucidated, particularly the dose- and frequency-dependent effects of irisin in cell cultures and in mouse models, extensive clinical evidence is emerging in support of its physiological relevance for bone and its role in secondary osteoporosis. In parallel, new studies identify irisin as a possible serum prognostic marker of bone pathologies [22,23].

In this review, we focus on recent findings about the involvement of irisin in different pathologies causing secondary osteoporosis.

## 2. Irisin in Primary Hyperparathyroidism

Primary hyperparathyroidism (PHPT) is an endocrine disease characterized by elevated calcium and PTH levels [24]. Affected patients develop a decreased BMD, particularly at the cortical site of the distal radius [25]. Joint pain is a common symptom in patients with PHPT [26,27], who, over time, develop osteoarthritis and osteoporosis [28]. Less frequent manifestations include Achilles tendon rupture, and sacral insufficiency fractures [26]. Biomolecular studies revealed that chronic high levels of PTH stimulate osteoclastogenesis indirectly by acting on osteoblasts. Indeed, PTH-stimulated osteoblasts secrete nuclear factor receptor-κB ligand (RANKL) and release low levels of OPG [25]. Emerging preclinical data regarding the possible interaction between PHT and irisin showed that, although in opposite ways, both affect bone, muscle, and adipose tissue. Therefore, recent studies focused on the cellular interaction between these two hormones by evaluating the expression of the irisin precursor, FNDC5, in skeletal muscle cells treated with 1-34 PTH (Teriparatide). Palermo et al. demonstrated that both short-term (3 h) and long-term (6 days) treatment with PTH negatively regulates the FNDC5 gene and protein expression in myotubes by acting through the PTH receptor, which in turn activates the phosphorylation of Erk1/2, most likely increasing intracellular cAMP [19]. The study also showed that irisin treatment decreases PTH receptor expression in osteoblasts, suggesting that this myokine may exert its anabolic effect on bone not only by stimulating osteoblast formation and function, but also by reducing the action of PTH on these cells [19]. Furthermore, serum irisin levels were lower in postmenopausal women with PHPT compared with control subjects [19]. This finding supported the results of other previous clinical investigations showing that irisin was inversely related to PTH in postmenopausal women with low bone mass [29] and in hemodialysis patients [30]. It is known that physical activity can help reduce PTH secretion, particularly if the exercise is chronic rather than resistance exercise [31]. This is relevant to the disease, as a slight reduction in circulating PTH levels may be desirable in patients with PHPT.

## 3. Irisin in Prader-Willi Syndrome (PWS)

Prader-Willi syndrome (PWS) is a rare genetic disorder that affects appetite, growth, the hormonal system, metabolism, cognitive function, and behavior. Most cases of PWS are attributed to a spontaneous genetic error that occurs due to a lack of gene expression in a specific part of the long arm of the paternal chromosome 15 [32]. The main mechanisms leading to the lack of gene expression responsible for Prader–Willi syndrome are interstitial deletion of the proximal long arm of chromosome 15 (del15q11-q13) (DEL15), maternal uniparental disomy of chromosome 15 (UPD15), and imprinting defects [33]. Patients with PWS show reduced muscle tone, short stature, incomplete sexual development, intellectual disability, peculiar behavior, poor nutrition, and initial failure to thrive, followed by hyperphagia and obesity in early childhood if eating is not controlled, multiple endocrine abnormalities, including growth hormone deficiency (GHD) and hypogonadism [34,35].

Notably, PWS patients also show bone defects. Children with PWS during puberty have normal bone mineral density (BMD) adjusted for reduced height [36,37,38], but in adolescence and adulthood, they show a decrease in total BMD and, in some cases, bone mineral content (BMC), because they have not reached bone mineral maturation; this is also due to pubertal delay/hypogonadism [39,40,41,42]. Consequently, osteoporosis is predominant in PWS individuals, who also have other orthopedic complications, worsened by weight gain, including scoliosis, kyphosis, hip dysplasia, flat feet, genu valgum, and fractures [41,43].

Many research groups showed an increasing interest in assessing irisin levels in adult and pediatric PWS patients in relation to genetic background, metabolic profile, cognitive impairment, and bone status. Hirsch et al. found increased amounts of salivary irisin in obese PWS compared with non-obese controls, whereas plasma irisin levels did not change significantly between the two groups [44,45]. Mai et al. also reported that PWS patients and controls had similar circulating irisin levels [46]. More recently, hypovitaminosis D was found by our group in a cohort of PWS patients [47]. Interestingly, irisin levels of those not supplemented with 25(OH)-Vitamin D were lower than levels detected in both controls and supplemented patients. Of note, a multiple regression analysis showed that irisin levels in both pediatric and adult PWS were predicted by genetic background and levels of 25(OH)-Vitamin D [47]. However, further studies are needed to understand the relationship between irisin and 25(OH)-Vitamin D and whether this interaction is influenced by disease type. Currently, there is limited and conflicting evidence about the effects of vitamin D on irisin synthesis. Preclinical studies in a diabetic rat model demonstrated that vitamin D supplementation upregulated FNDC5 gene expression in muscle but not serum irisin levels [48]. Studies conducted in healthy young adults showed that a single 100,000 IU dose of vitamin D did not influence irisin levels [49]. However, in older adults affected by type 2 diabetes mellitus with vitamin D deficiency, 8 weeks of vitamin D supplementation (50,000 IU/week) were effective for increasing irisin levels [50].

In conclusion, although studies to date have not found differences in circulating levels of irisin in PWS patients compared with matched controls, a possible role of genetic background in PWS on irisin level has emerged. In addition, further studies are desirable to evaluate whether vitamin D supplementation may be a key factor in the regulation of circulating irisin levels.

## 4. Irisin in GH Deficiency (GHD)

Growth hormone deficiency (GHD), also known as dwarfism or pituitary dwarfism, is a condition caused by insufficient amounts of growth hormone in the body. Children with GHD have an abnormally short stature with normal body proportions and were shown to have low BMD [51]. In adults, GHD causes abnormalities in body composition, problems with movement and exercise in conjunction with decreased BMD, and increased fracture risk [52,53]. GHD can be present at birth (congenital) or develop later (acquired).

In the pediatric population, a reduction in irisin may play a role in the pathogenesis of childhood obesity [54] because this myokine plays a very important role in the regulation of adipose tissue metabolism [55,56] and correlates with glucose tolerance and insulin resistance in humans [57,58]. Several studies showed a possible link between skeletal muscle and adipose tissue mediated by irisin. Irisin plays a role in both adipose tissue and glucose metabolism; therefore, changes in irisin may mediate the effects of GH deficiency (GHD) and GH replacement (GHR) on these endpoints.

Specifically, GHD is characterized by altered body composition with reduced muscle mass and increased adiposity [58,59], as well as metabolic alterations [60,61], and GHR may cause a reversal of these effects [62,63]. A direct interaction between irisin and GH was documented in nonmammalian species. Indeed, fish irisin acts directly at the level of the pituitary gland to inhibit the expression of GH transcription through multiple signaling pathways [64]. However, it is not known whether GHD and GHR can affect irisin levels or whether changes in irisin levels in GHD and its replacement are associated with changes in body composition and glucose homeostasis. A previous study assessed circulating irisin levels in a cohort of children with GHD at diagnosis, with a bone age delay of at least 1 year from the chronologic age, as estimated by radiography of the left wrist and hand. Changes of irisin levels during GHR, and any association of irisin with body composition and metabolic parameters, were also assessed. The results showed that GHD is associated with lower irisin levels, in turn associated with changes in body composition and metabolic endpoints [65]. After 12 months of GHR, children with GHD showed a significant increase in serum irisin levels, along with an increase in IGF-I, and an improvement in bone/chronological age ratio. In agreement with this finding, a previous study, performed in patients with Turner syndrome, showed an increase in irisin, concomitant with an increase in IGF-I levels, after the administration of supraphysiological doses of GHR (0.05 mg/kg/d) [66]. Therefore, this suggests that future studies will be needed to understand whether the association between GH and irisin is causative or not, and it would be interesting to evaluate whether, in GHD patients after GHR, increasing irisin levels can improve musculoskeletal homeostasis, and can revert the bone age delay in children.

## 5. Irisin in Cushing’s Disease (CD)

Cushing’s disease (CD) is a rare condition that results from an excess of cortisol in the body. Cortisol is a hormone normally produced by the adrenal glands and is essential for life. Excess cortisol may be caused by a pituitary tumor that secretes ACTH. However, CD generated by this oversecretion of ACTH in the pituitary gland differs from other causes of Cushing’s syndrome, i.e., adrenal overproduction of cortisol or paraneoplastic ectopic production of ACTH. When left untreated, CD leads to excess mortality [67]. However, there is uncertainty about the long-term survival of patients with CD in remission, because it causes metabolic, psychiatric, cardiovascular, and musculoskeletal comorbidities associated with hypercortisolism [68,69]. Glucocorticoid levels affect skeletal muscle activity, resulting in muscle atrophy and weakness in patients with CD. In fact, 40–70% of patients with CD report muscle weakness especially in the proximal muscles of the lower extremities [70,71]. Therefore, these patients have difficulty getting up from a squatting position or climbing stairs, whereas they have less difficulty running or walking [72,73]. Moreover, patients with CD, develop sarcopenia, which is generally associated with obesity and osteoporosis, leading to a condition called osteosarcopenic obesity [73].

Excess cortisol could affect circulating irisin levels, especially since skeletal muscle, the main source of irisin, is one of the target organs of cortisol. A recent study evaluated circulating irisin levels in patients with active, controlled Cushing’s disease [73]. Guarnotta et al., observed that circulating irisin levels were lower in patients with CD before and after the correction of hypercortisolism compared with controls [73]. Given the role of irisin as a player in the bone, muscle, and adipose tissue axis, further studies will certainly be relevant to understand whether this molecule represents a marker for the diagnosis of osteosarcopenia and central obesity in patients with CD.

## 6. Irisin in Inflammatory Bowel Disease (IBD)

Inflammatory bowel disease (IBD) is characterized by chronic inflammation of the intestine and gastrointestinal disorders; the most common forms of IBD are Crohn’s disease (CD) and ulcerative colitis (UC) [74]. Patients with IBD suffer not only from intestinal pathology but also from multiple comorbidities induced by extraintestinal inflammation. Malnutrition, vitamin D deficiency, reduced physical activity, hypogonadism, delayed puberty, inflammation, and corticosteroid use are common findings in patients with IBD and, in turn, may negatively impact the skeletal system [75]. Indeed, IBD causes osteopenia, osteoporosis, and a subsequently high fracture risk [76,77]. In these patients, the bone microstructure is damaged. Inflammation generates a decrease in bone mass because it increases osteocyte protein expression and osteoclast activity; it also increases the expression of the receptor activator of NF-kB ligand (RANKL), TNF-a, and IL-6, which are factors implicated in IBD and known to stimulate bone resorption [78]. However, it is not known whether the immunological processes that drive bone loss in IBD are distinct from or parallel to those in the intestine. There is currently no cure for chronic IBD, so all efforts are being made for treatments aimed at mitigating the symptoms of the disease [79]. Given the emerging anti-inflammatory role of irisin, a recent study examined how exogenous treatment with irisin might improve disease status in a rat model of TNBS-induced chronic IBD [80]. This rat model is characterized by morphological and functional changes in the gut that occur in parallel in the bone, with increased bone resorption and decreased bone formation. Results showed that exogenous treatment with irisin blocked the gut from inflammatory changes, improved lymphatic structure, and recovered bone turnover by reducing TNF-a and RANKL [80], thus proposing irisin as a promising clinical approach in chronic inflammatory conditions [80]. Although information on irisin and IBD in humans is currently unknown, it is well recognized that moderate exercise may exert an ameliorative effect in this disease. Crohn’s disease patients who exercised were significantly less likely to develop active disease after six months. In addition, moderate exercise was shown to exert a positive effect on weight maintenance and delay of osteoporosis in patients with IBD [81]. Conversely, it is known that, depending on its intensity and duration, exercise can cause mild transient systemic inflammation and increases the release of pro-inflammatory cytokines, thereby exacerbating gastrointestinal symptoms. Therefore, studies correlating serum irisin levels with inflammatory cytokines in IBD are not only desirable but also urgent since physical activity is one of the strongly recommended nonpharmacological therapies for the mental and physical well-being of these patients.

## 7. Conclusions

Secondary osteoporosis most commonly affects patients including men and patients without classic clinical risk factors. More than 50% of premenopausal women and between 50% and 80% of men have secondary osteoporosis, whose skeletal fragility may result from the underlying chronic condition that can either interfere with the achievement of peak bone mass during growth or increase the rate of bone loss due to the chronicity of the condition. Unidentified secondary osteoporosis may contribute to the severity of osteoporosis or inadequate responses to treatment. For example, in glucocorticoid-induced osteoporosis, if diagnosed early, the use of an anabolic agent may be preferable. Therefore, it is critical to identify new biomarkers involved in bone loss caused by secondary osteoporosis in order to diagnose it at the earliest possible time. Although there are still few studies, emerging data showing altered circulating levels of irisin in secondary osteoporosis are promising. Further studies are needed to understand whether the modulation of irisin is caused by mechanisms underlying the disease itself, e.g., genetic causes, or whether it is the muscle and bone damage, hallmarks of these diseases, that influence the circulating levels of this myokine.

## References

[1] Khajuria D.K., Razdan R., Mahapatra D.R. (2011). Drugs for the management of osteoporosis: A review. Rev. Bras. Reumatol..

[2] Curtis E., Litwic A., Cooper C., Dennison E. (2015). Determinants of Muscle and Bone Aging. J. Cell Physiol..

[3] Boström P., Wu J., Jedrychowski M.P., Korde A., Ye L., Lo J.C., Rasbach K.A., Boström E.A., Choi J.H., Long J.Z. (2012). A PGC1-α-dependent myokine that drives brown-fat-like development of white fat and thermogenesis. Nature.

[4] Eckardt K., Görgens S.W., Raschke S., Eckel J. (2014). Myokines in insulin resistance and type 2 diabetes. Diabetologia.

[5] Kim O.Y., Song J. (2018). The Role of Irisin in Alzheimer’s Disease. J. Clin. Med..

[6] Peng H., Wang Q., Lou T., Qin J., Jung S., Shetty V., Li F., Wang Y., Feng X.H., Mitch W.E. (2017). Myokine mediated muscle-kidney crosstalk suppresses metabolic reprogramming and fibrosis in damaged kidneys. Nat. Commun..

[7] Buccoliero C., Oranger A., Colaianni G., Pignataro P., Zerlotin R., Lovero R., Errede M., Grano M. (2021). The effect of Irisin on bone cells in vivo and in vitro. Biochem. Soc. Trans..

[8] Colucci S., Colaianni G., Brunetti G., Ferranti F., Mascetti G., Mori G., Grano M. (2020). Irisin prevents microgravity-induced impairment of osteoblast differentiation in vitro during the space flight CRS-14 mission. FASEB J..

[9] Colaianni G., Cuscito C., Mongelli T., Oranger A., Mori G., Brunetti G., Colucci S., Cinti S., Grano M. (2014). Irisin enhances osteoblast differentiation in vitro. Int. J. Endocrinol..

[10] Colaianni G., Cuscito C., Mongelli T., Pignataro P., Buccoliero C., Liu P., Lu P., Sartini L., Di Comite M., Mori G. (2015). The myokine irisin increases cortical bone mass. Proc. Natl. Acad. Sci. USA.

[11] Colaianni G., Mongelli T., Cuscito C., Pignataro P., Lippo L., Spiro G., Notarnicola A., Severi I., Passeri G., Mori G. (2017). Irisin prevents and restores bone loss and muscle atrophy in hind-limb suspended mice. Sci. Rep..

[12] Estell E.G., Le P.T., Vegting Y., Kim H., Wrann C., Bouxsein M.L., Nagano K., Baron R., Spiegelman B.M., Rosen C.J. (2020). Irisin directly stimulates osteoclastogenesis and bone resorption in vitro and in vivo. Elife.

[13] Kim H., Wrann C.D., Jedrychowski M., Vidoni S., Kitase Y., Nagano K., Zhou C., Chou J., Parkman V.A., Novick S.J. (2018). Irisin Mediates Effects on Bone and Fat via αV Integrin Receptors. Cell.

[14] Zhang J., Valverde P., Zhu X., Murray D., Wu Y., Yu L., Jiang H., Dard M.M., Huang J., Xu Z. (2017). Exercise-induced irisin in bone and systemic irisin administration reveal new regulatory mechanisms of bone metabolism. Bone Res..

[15] Silva B.C., Bilezikian J.P. (2015). Parathyroid hormone: Anabolic and catabolic actions on the skeleton. Curr. Opin. Pharmacol..

[16] Storlino G., Colaianni G., Sanesi L., Lippo L., Brunetti G., Errede M., Colucci S., Passeri G., Grano M. (2020). Irisin Prevents Disuse-Induced Osteocyte Apoptosis. J. Bone Miner. Res..

[17] Colucci S.C., Buccoliero C., Sanesi L., Errede M., Colaianni G., Annese T., Khan M.P., Zerlotin R., Dicarlo M., Schipani E. (2021). Systemic Administration of Recombinant Irisin Accelerates Fracture Healing in Mice. Int. J. Mol. Sci..

[18] Faienza M.F., Brunetti G., Sanesi L., Colaianni G., Celi M., Piacente L., D’Amato G., Schipani E., Colucci S., Grano M. (2018). High irisin levels are associated with better glycemic control and bone health in children with Type 1 diabetes. Diabetes Res. Clin. Pract..

[19] Palermo A., Sanesi L., Colaianni G., Tabacco G., Naciu A.M., Cesareo R., Pedone C., Lelli D., Brunetti G., Mori G. (2019). A Novel Interplay Between Irisin and PTH: From Basic Studies to Clinical Evidence in Hyperparathyroidism. J. Clin. Endocrinol. Metab..

[20] Colaianni G., Errede M., Sanesi L., Notarnicola A., Celi M., Zerlotin R., Storlino G., Pignataro P., Oranger A., Pesce V. (2021). Irisin Correlates Positively With BMD in a Cohort of Older Adult Patients and Downregulates the Senescent Marker p21 in Osteoblasts. J. Bone Miner. Res..

[21] Farr J.N., Khosla S. (2019). Cellular senescence in bone. Bone.

[22] Yan J., Liu H.J., Guo W.C., Yang J. (2018). Low serum concentrations of Irisin are associated with increased risk of hip fracture in Chinese older women. Joint. Bone Spine.

[23] Mao Y., Xu W., Xie Z., Dong Q. (2016). Association of Irisin and CRP Levels with the Radiographic Severity of Knee Osteoarthritis. Genet. Test. Mol. Biomark..

[24] Silva B.C., Cusano N.E., Bilezikian J.P. (2018). Primary hyperparathyroidism. Best Pract. Res. Clin. Endocrinol. Metab..

[25] Silva B.C., Costa A.G., Cusano N.E., Kousteni S., Bilezikian J.P. (2011). Catabolic and anabolic actions of parathyroid hormone on the skeleton. J. Endocrinol. Invest..

[26] Pappu R., Jabbour S.A., Reginato A.M., Reginato A.J. (2016). Musculoskeletal manifestations of primary hyperparathyroidism. Clin. Rheumatol..

[27] Murray S.E., Pathak P.R., Pontes D.S., Schneider D.F., Schaefer S.C., Chen H., Sippel R.S. (2013). Timing of symptom improvement after parathyroidectomy for primary hyperparathyroidism. Surgery.

[28] Chiodini I., Cairoli E., Palmieri S., Pepe J., Walker M.D. (2018). Non classical complications of primary hyperparathyroidism. Best Pract. Res. Clin. Endocrinol. Metab..

[29] Anastasilakis A.D., Polyzos S.A., Makras P., Gkiomisi A., Bisbinas I., Katsarou A., Filippaios A., Mantzoros C.S. (2014). Circulating irisin is associated with osteoporotic fractures in postmenopausal women with low bone mass but is not affected by either teriparatide or denosumab treatment for 3 months. Osteoporos. Int..

[30] He L., He W.Y., A L.T., Yang W.L., Zhang A.H. (2018). Lower Serum Irisin Levels Are Associated with Increased Vascular Calcification in Hemodialysis Patients. Kidney Blood Press Res..

[31] Lombardi G., Ziemann E., Banfi G., Corbetta S. (2020). Physical Activity-Dependent Regulation of Parathyroid Hormone and Calcium-Phosphorous Metabolism. Int. J. Mol. Sci..

[32] Tauber M., Diene G. (2021). Prader-Willi syndrome: Hormone therapies. Handb. Clin. Neurol..

[33] Butler M.G., Hartin S.N., Hossain W.A., Manzardo A.M., Kimonis V., Dykens E., Gold J.A., Kim S.J., Weisensel N., Tamura R. (2019). Molecular genetic classification in Prader-Willi syndrome: A multisite cohort study. J. Med. Genet..

[34] Angulo M.A., Butler M.G., Cataletto M.E. (2015). Prader-Willi syndrome: A review of clinical, genetic, and endocrine findings. J. Endocrinol. Invest..

[35] Driscoll D.J., Miller J.L., Schwartz S., Cassidy S.B., Adam M.P., Ardinger H.H., Pagon R.A., Wallace S.E., Bean L.J.H., Mirzaa G., Amemiya A. (2012). Prader-Willi Syndrome. GeneReviews(®).

[36] De Lind van Wijngaarden R.F., Festen D.A., Otten B.J., van Mil E.G., Rotteveel J., Odink R.J., van Leeuwen M., Haring D.A., Bocca G., Mieke Houdijk E.C. (2009). Bone mineral density and effects of growth hormone treatment in prepubertal children with Prader-Willi syndrome: A randomized controlled trial. J. Clin. Endocrinol. Metab..

[37] Edouard T., Deal C., Van Vliet G., Gaulin N., Moreau A., Rauch F., Alos N. (2012). Muscle-bone characteristics in children with Prader-Willi syndrome. J. Clin. Endocrinol. Metab..

[38] Van Mil E.G., Westerterp K.R., Gerver W.J., Van Marken Lichtenbelt W.D., Kester A.D., Saris W.H. (2001). Body composition in Prader-Willi syndrome compared with nonsyndromal obesity: Relationship to physical activity and growth hormone function. J. Pediatr..

[39] Vestergaard P., Kristensen K., Bruun J.M., Østergaard J.R., Heickendorff L., Mosekilde L., Richelsen B. (2004). Reduced bone mineral density and increased bone turnover in Prader-Willi syndrome compared with controls matched for sex and body mass index—A cross-sectional study. J. Pediatr..

[40] Butler M.G., Haber L., Mernaugh R., Carlson M.G., Price R., Feurer I.D. (2001). Decreased bone mineral density in Prader-Willi syndrome: Comparison with obese subjects. Am. J. Med. Genet..

[41] Höybye C., Hilding A., Jacobsson H., Thorén M. (2002). Metabolic profile and body composition in adults with Prader-Willi syndrome and severe obesity. J. Clin. Endocrinol. Metab..

[42] Brunetti G., Grugni G., Piacente L., Delvecchio M., Ventura A., Giordano P., Grano M., D’Amato G., Laforgia D., Crinò A. (2018). Analysis of Circulating Mediators of Bone Remodeling in Prader-Willi Syndrome. Calcif. Tissue Int..

[43] Van Nieuwpoort I.C., Twisk J.W.R., Curfs L.M.G., Lips P., Drent M.L. (2018). Body composition, adipokines, bone mineral density and bone remodeling markers in relation to IGF-1 levels in adults with Prader-Willi syndrome. Int. J. Pediatr. Endocrinol..

[44] Hirsch H.J., Gross I., Pollak Y., Eldar-Geva T., Gross-Tsur V. (2015). Irisin and the Metabolic Phenotype of Adults with Prader-Willi Syndrome. PLoS ONE.

[45] Hirsch H.J., Gross-Tsur V., Sabag Y., Nice S., Genstil L., Benarroch F., Constantini N. (2020). Myokine levels after resistance exercise in young adults with Prader-Willi syndrome (PWS). Am. J. Med. Genet. A.

[46] Mai S., Grugni G., Mele C., Vietti R., Vigna L., Sartorio A., Aimaretti G., Scacchi M., Marzullo P. (2020). Irisin levels in genetic and essential obesity: Clues for a potential dual role. Sci. Rep..

[47] Faienza M.F., Brunetti G., Grugni G., Fintini D., Convertino A., Pignataro P., Crinò A., Colucci S., Grano M. (2021). The genetic background and vitamin D supplementation can affect irisin levels in Prader-Willi syndrome. J. Endocrinol. Invest..

[48] Nadimi H., Djazayery A., Javanbakht M.H., Dehpour A., Ghaedi E., Derakhshanian H., Mohammadi H., Zarei M., Djalali M. (2019). The Effect of Vitamin D Supplementation on Serum and Muscle Irisin Levels, and FNDC5 Expression in Diabetic Rats. Rep. Biochem. Mol. Biol..

[49] Cavalier É., Mismetti V., Souberbielle J.C. (2014). Evaluation of circulating irisin levels in healthy young individuals after a single 100,000 IU vitamin D dose. Ann. Endocrinol..

[50] Safarpour P., Daneshi-Maskooni M., Vafa M., Nourbakhsh M., Janani L., Maddah M., Amiri F.S., Mohammadi F., Sadeghi H. (2020). Vitamin D supplementation improves SIRT1, Irisin, and glucose indices in overweight or obese type 2 diabetic patients: A double-blind randomized placebo-controlled clinical trial. BMC Fam. Pract..

[51] Baroncelli G.I., Bertelloni S., Sodini F., Saggese G. (2003). Acquisition of bone mass in normal individuals and in patients with growth hormone deficiency. J. Pediatr. Endocrinol. Metab..

[52] Carroll P.V., Christ E.R., Bengtsson B.A., Carlsson L., Christiansen J.S., Clemmons D., Hintz R., Ho K., Laron Z., Sizonenko P. (1998). Growth hormone deficiency in adulthood and the effects of growth hormone replacement: A review. Growth Hormone Research Society Scientific Committee. J. Clin. Endocrinol. Metab..

[53] Holmes S.J., Economou G., Whitehouse R.W., Adams J.E., Shalet S.M. (1994). Reduced bone mineral density in patients with adult onset growth hormone deficiency. J. Clin. Endocrinol. Metab..

[54] Pyrżak B., Demkow U., Kucharska A.M. (2015). Brown Adipose Tissue and Browning Agents: Irisin and FGF21 in the Development of Obesity in Children and Adolescents. Adv. Exp. Med. Biol..

[55] Moreno-Navarrete J.M., Ortega F., Serrano M., Guerra E., Pardo G., Tinahones F., Ricart W., Fernández-Real J.M. (2013). Irisin is expressed and produced by human muscle and adipose tissue in association with obesity and insulin resistance. J. Clin. Endocrinol. Metab..

[56] Aydin S. (2014). Three new players in energy regulation: Preptin, adropin and irisin. Peptides.

[57] Huh J.Y., Panagiotou G., Mougios V., Brinkoetter M., Vamvini M.T., Schneider B.E., Mantzoros C.S. (2012). FNDC5 and irisin in humans: I. Predictors of circulating concentrations in serum and plasma and II. mRNA expression and circulating concentrations in response to weight loss and exercise. Metabolism.

[58] Al-Daghri N.M., Alkharfy K.M., Rahman S., Amer O.E., Vinodson B., Sabico S., Piya M.K., Harte A.L., McTernan P.G., Alokail M.S. (2014). Irisin as a predictor of glucose metabolism in children: Sexually dimorphic effects. Eur. J. Clin. Invest..

[59] Matusik P., Klesiewicz M., Klos K., Stasiulewicz M., Barylak A., Nazarkiewicz P., Malecka-Tendera E. (2016). Baseline Body Composition in Prepubertal Short Stature Children with Severe and Moderate Growth Hormone Deficiency. Int. J. Endocrinol..

[60] Lanes R., Soros A., Gunczler P., Paoli M., Carrillo E., Villaroel O., Palacios A. (2006). Growth hormone deficiency, low levels of adiponectin, and unfavorable plasma lipid and lipoproteins. J. Pediatr..

[61] Ciresi A., Amato M.C., Criscimanna A., Mattina A., Vetro C., Galluzzo A., D’Acquisto G., Giordano C. (2007). Metabolic parameters and adipokine profile during GH replacement therapy in children with GH deficiency. Eur. J. Endocrinol..

[62] Elbornsson M., Götherström G., Bosæus I., Bengtsson B., Johannsson G., Svensson J. (2013). Fifteen years of GH replacement improves body composition and cardiovascular risk factors. Eur. J. Endocrinol..

[63] Ciresi A., Guarnotta V., Pizzolanti G., Giordano C. (2018). Comparison between euglycemic hyperinsulinemic clamp and surrogate indices of insulin sensitivity in children with growth hormone deficiency. Growth Horm. IGF Res..

[64] Lian A., Li X., Jiang Q. (2017). Irisin inhibition of growth hormone secretion in cultured tilapia pituitary cells. Mol. Cell Endocrinol..

[65] Ciresi A., Pizzolanti G., Guarnotta V., Giordano C. (2019). Circulating Irisin Levels in Children With GH Deficiency Before and After 1 Year of GH Treatment. J. Clin. Endocrinol. Metab..

[66] Wikiera B., Zawadzka K., Łaczmański Ł., Słoka N., Bolanowski M., Basiak A., Noczyńska A., Daroszewski J. (2017). Growth Hormone Treatment Increases Plasma Irisin Concentration in Patients with Turner Syndrome. Horm. Metab. Res..

[67] Baussart B., Gaillard S. (2021). Pituitary surgery for Cushing’s disease. Acta Neurochir..

[68] Pivonello R., De Martino M.C., Iacuaniello D., Simeoli C., Muscogiuri G., Carlomagno F., De Leo M., Cozzolino A., Colao A. (2016). Metabolic Alterations and Cardiovascular Outcomes of Cortisol Excess. Front. Horm. Res..

[69] Giordano C., Guarnotta V., Pivonello R., Amato M.C., Simeoli C., Ciresi A., Cozzolino A., Colao A. (2014). Is diabetes in Cushing’s syndrome only a consequence of hypercortisolism?. Eur. J. Endocrinol..

[70] Bolland M.J., Holdaway I.M., Berkeley J.E., Lim S., Dransfield W.J., Conaglen J.V., Croxson M.S., Gamble G.D., Hunt P.J., Toomath R.J. (2011). Mortality and morbidity in Cushing’s syndrome in New Zealand. Clin. Endocrinol..

[71] Guarnotta V., Amato M.C., Pivonello R., Arnaldi G., Ciresi A., Trementino L., Citarrella R., Iacuaniello D., Michetti G., Simeoli C. (2017). The degree of urinary hypercortisolism is not correlated with the severity of cushing’s syndrome. Endocrine.

[72] Minetto M.A., Lanfranco F., Motta G., Allasia S., Arvat E., D’Antona G. (2011). Steroid myopathy: Some unresolved issues. J. Endocrinol. Investig..

[73] Guarnotta V., Prinzi A., Pitrone M., Pizzolanti G., Giordano C. (2020). Circulating Irisin Levels as a Marker of Osteosarcopenic-Obesity in Cushing’s Disease. Diabetes Metab. Syndr. Obes..

[74] Yang G.Y., Taboada S., Liao J. (2009). Inflammatory bowel disease: A model of chronic inflammation-induced cancer. Methods Mol. Biol..

[75] Szafors P., Che H., Barnetche T., Morel J., Gaujoux-Viala C., Combe B., Lukas C. (2018). Risk of fracture and low bone mineral density in adults with inflammatory bowel diseases. A systematic literature review with meta-analysis. Osteoporos Int..

[76] Soare I., Sirbu A., Martin S., Diculescu M., Mateescu B., Tieranu C., Fica S. (2021). Assessment of bone quality with trabecular bone score in patients with inflammatory bowel disease. Sci. Rep..

[77] Ghishan F.K., Kiela P.R. (2011). Advances in the understanding of mineral and bone metabolism in inflammatory bowel diseases. Am. J. Physiol.-Gastrointest. Liver Physiol..

[78] Metzger C.E., Narayanan A., Zawieja D.C., Bloomfield S.A. (2017). Inflammatory Bowel Disease in a Rodent Model Alters Osteocyte Protein Levels Controlling Bone Turnover. J. Bone Miner. Res..

[79] Seibel M.J., Cooper M.S., Zhou H. (2013). Glucocorticoid-induced osteoporosis: Mechanisms, management, and future perspectives. Lancet Diabetes Endocrinol..

[80] Narayanan S.A., Metzger C.E., Bloomfield S.A., Zawieja D.C. (2018). Inflammation-induced lymphatic architecture and bone turnover changes are ameliorated by irisin treatment in chronic inflammatory bowel disease. FASEB J..

[81] Bilski J., Mazur-Bialy A., Brzozowski B., Magierowski M., Zahradnik-Bilska J., Wójcik D., Magierowska K., Kwiecien S., Mach T., Brzozowski T. (2016). Can exercise affect the course of inflammatory bowel disease? Experimental and clinical evidence. Pharmacol. Rep..

